# Therapeutic Targeting of SGLT2: A New Era in the Treatment of Diabetes and Diabetic Kidney Disease

**DOI:** 10.3389/fendo.2021.749010

**Published:** 2021-11-01

**Authors:** James Shaffner, Bohan Chen, Deepak K. Malhotra, Lance D. Dworkin, Rujun Gong

**Affiliations:** Department of Medicine, Division of Nephrology, University of Toledo College of Medicine, Toledo, OH, United States

**Keywords:** diabetes, diabetic kidney disease, glycosuria, ketosis, SGLT2

## Abstract

As the prevalence of diabetic kidney disease (DKD) continues to rise, so does the need for a novel therapeutic modality that can control and slow its progression to end-stage renal disease. The advent of sodium-glucose cotransporter-2 (SGLT2) inhibitors has provided a major advancement for the treatment of DKD. However, there still remains insufficient understanding of the mechanism of action and effectiveness of this drug, and as a result, its use has been very limited. Burgeoning evidence suggests that the SGLT2 inhibitors possess renal protective activities that are able to lower glycemic levels, improve blood pressure/hemodynamics, cause bodyweight loss, mitigate oxidative stress, exert anti-inflammatory and anti-fibrotic effects, reduce urinary albumin excretion, lower uric acid levels, diminish the activity of intrarenal renin-angiotensin-aldosterone system, and reduce natriuretic peptide levels. SGLT2 inhibitors have been shown to be safe and beneficial for use in patients with a GFR ≥30mL/min/1.73m^2^, associated with a constellation of signs of metabolic reprogramming, including enhanced ketogenesis, which may be responsible for the correction of metabolic reprogramming that underlies DKD. This article aims to provide a comprehensive overview and better understanding of the SGLT2 inhibitor and its benefits as it pertains to renal pathophysiology. It summarizes our recent understanding on the mechanisms of action of SGLT2 inhibitors, discusses the effects of SGLT2 inhibitors on diabetes and DKD, and presents future research directions and therapeutic potential.

## Introduction

Glucose (C_6_H_12_O_6_) is a monosaccharide or “simple sugar”, meaning that it is the most basic unit of carbohydrate and cannot be further hydrolyzed to a simpler chemical compound. It is the major source of fuel in the human body and comes in various forms such as monosaccharides, disaccharides and polysaccharides, such as starch. The body maintains fasting glucose levels at around 80mg/dL in order to provide a continuous supply of glucose to tissues that are unable to synthesize glucose on their own, such as brain and red blood cells. In order to provide the body with a continuous source of glucose during the fasting state, it is stored in the form of glycogen with the most abundant stores located in the liver and skeletal muscle. It is also synthesized endogenously in the liver and kidney from non-carbohydrate sources by gluconeogenesis. These precursors include lactate, glycerol, alanine, and glutamine. The kidney primarily uses lactate, glutamine, and glycerol with lactate being the largest source of substrate for gluconeogenesis in the kidney (1, 2).

A critical step in the utilization of glucose is its transport across cell membranes. There are two major classes of glucose transport proteins: the glucose transporters (GLUTs) and sodium-dependent glucose transporters (SGLTs). GLUTs are members of the solute carrier family 2A *(SLC2A)* gene family and utilize facilitated diffusion to transport glucose across the cell membrane. There are currently 14 GLUT genes identified in the human body and are located in fetal tissues, erythrocytes, blood-brain barrier, neurons, adipose tissue, striated muscle, testes, small intestine epithelium, liver cells, pancreatic beta cells, and renal tubular cells (3). In particular, GLUT2 allows glucose to be transported across the basolateral membrane of the kidney and mutations of this protein are responsible for Fanconi-Bickel Syndrome, a rare type of glycogen storage disease characterized by the accumulation of glycogen in the liver and kidney (4). SGLTs are cotransporter proteins of the *SLC5A* gene family which bind both sodium and glucose for cotransport across the cell membrane, utilizing the sodium electrochemical gradient maintained by the Na^+^/K^+^ ATPase. There are currently 6 SGLT isoforms identified as SGLT1, SGLT2, SGLT3, SGLT4, SGLT5, and SGLT6 **(** **Table 1** **).** SGLT1 was the first identified and the most extensively studied SGLT and is located primarily in the intestine and the S3 segment of the proximal convoluted tubule (PCT) where it reabsorbs 10-20% of the glucose that escapes uptake by SGLT2. SGLT3 is expressed in the enteric neurons and is believed to be a glucose sensor (5, 6). The study of the SGLT family of transporters is ongoing and there are still many unanswered questions that remain elusive. This review will focus on the SGLT2 and cover the latest research and development as it pertains to renal pathobiology.

**Table 1:** Expression of different SGLT isoforms in various mammalian organ systems and their selective inhibitors.

Transporter	SGLT1	SGLT2	SGLT3	SGLT4	SGLT5	SGLT6
**Gene name**	*SLC5A1*	*SLC5A2*	*SLC5A4*	*SLC5A9*	*SLC5A10*	*SLC5A11*
**Tissue distribution**	■ Intestine ■ Kidney ■ Trachea ■ Heart ■ Brain ■ Testis ■ Prostate	■ Kidney ■ Pancreas ■ Liver ■ Thyroid ■ Muscle ■ Heart	■ Intestine ■ Testis ■ Uterus ■ Lung ■ Brain ■ Thyroid	■ Intestine ■ Kidney ■ Liver ■ Brain ■ Trachea ■ Lung ■ Uterus ■ Pancreas	■ Kidney Cortex	■ Intestine ■ Kidney Cortex ■ Brain ■ Spinal Cord
** Endogenous substrates**	■ D-glucose ■ D-galactose	■ Glucose	■ D-glucose	■ D-mannose ■ D-glucose	■ D-glucose ■ Galactose	■ D-chiro-inositol ■ D-glucose
** Inhibitors**	■ Phlorizin ■ KGA 2727 ■ LX4211 ■ GSK-1614235	■ Phlorizin ■ Dapagliflozin ■ Canagliflozin ■ Empagliflozin ■ Ipragliflozin ■ Luseogliflozin ■ Tofogliflozin ■ Ertugliflozin ■ Remogliflozin ■ Sotagliflozin	■ Phlorizin	■ Phlorizin	■ Phlorizin ■ Gliflozins	■ Glucose ■ Canagliflozin ■ Dapagliflozin ■ Phlorizin ■ Cpd B

## Pathobiology of SGLT2

The SGLT2 is a member of the *SLC5A* gene family, part of the Sodium Solute Symporter (SSS) superfamily of proteins and capable of transporting glucose. The SGLT2 transport protein *(SLC5A2)* is the transporter responsible for 80-90% of glucose reabsorption located and abundantly expressed on the brush border membrane of the S1-S2 segments of the early proximal convoluted tubule of the kidney (7). Additionally, there are reports suggesting that SGLT2 is also expressed in the liver, pancreas, heart, thyroid, and muscle (8, 9). Vallon et al. were the first to report the immunolocation of SGLT2 in the mouse kidney (10). Using immunohistochemical analysis with an SGLT2 specific antibody, they demonstrated that the transport protein was localized to the apical brush border membrane of the early proximal tubule in wild-type (WT) mice where it was responsible for reabsorption of all the filtered glucose. The specificity of the immunohistochemical staining was confirmed by comparing WT to transgenic SGLT2 null mice (SGLT2^-/-^) (10). This distribution of SGLT2 was subsequently replicated in human kidneys by immunohistochemistry, though no physiological studies on isolated tubules from human kidneys have been performed (11).

Although the atomic structure of human SGLT (hSGLT) has not yet been established, the structure of a bacterial homolog Vibrio parahaemolyticis (vSGLT) with 32% amino acid identity to hSGLT1 has been solved and refined to 2.7 A (8, 12). The first structure of vSGLT was solved in collaboration with Abramson and his team (12). The functional properties show that vSGLT has much in common with hSGLT1, but there are differences in sugar selectivity and Na^+^-to-sugar transport stoichiometry which more resembles SGLT2 (1:1 rather than the 2:1 for SGLT1). Relative to human SGLT1, there is between 50-70% identity and 67-84% similarity in the sequences for SGLT2 (8). The amino acid sequence of SGLT2 is believed to resemble a 14-transmembrane helix model, but this has yet to be validated.

The SGLT2 is a high-capacity and low-affinity co-transporter located at the apical brush border of the PCT extending from the S1 to S2 segments. Located at the basolateral membrane of the PCT is the Na^+^/K^+^ ATPase pump which creates the intracellular Na^+^ electrochemical gradient needed for the operation of the SGLT2 to drive the transport of Na^+^ and glucose into the cells. Glucose moves against its electrochemical gradient following sodium transportation along its gradient in 1:1 stoichiometry. The intracellular glucose is then diffused to the interstitium through GLUTs located on the basolateral membrane of the PCT cells.

In the fasting (postabsorptive) state in healthy individuals, the kidney contributes around 20-25% of the glucose released into the circulation by gluconeogenesis (15–55 g/day), with the liver responsible for the remainder *via* both glycogenolysis and gluconeogenesis (13). Renal gluconeogenesis occurs primarily in the PCT cells located in the renal cortex and is regulated by insulin and catecholamines. Insulin decreases renal gluconeogenesis and its substrates lactate, glutamine, and glycerol, whereas catecholamines stimulate renal gluconeogenesis and its substrates, reduce renal glucose uptake, and decrease insulin release (2, 14–16). In type 2 diabetes mellitus (T2DM), gluconeogenesis is increased in both kidney and liver with increases up to 300% and 30% respectively.

In the postprandial state, gluconeogenesis increases renal glucose release more than twofold accounting for ~60% of the endogenous glucose release during the 4-6 hours period following meals. The mechanism is unknown but thought to allow for repletion of hepatic glycogen stores by permitting suppression of hepatic glucose release (13). In T2DM this glucose release is estimated to be ~30% higher when compared to healthy individuals. As a result, insulin resistance increases and suppression of renal glucose release decreases, which in turn causes an upregulation of GLUTs (1).

## Targeting of SGLT2 in Diabetes

**Figure 1:**
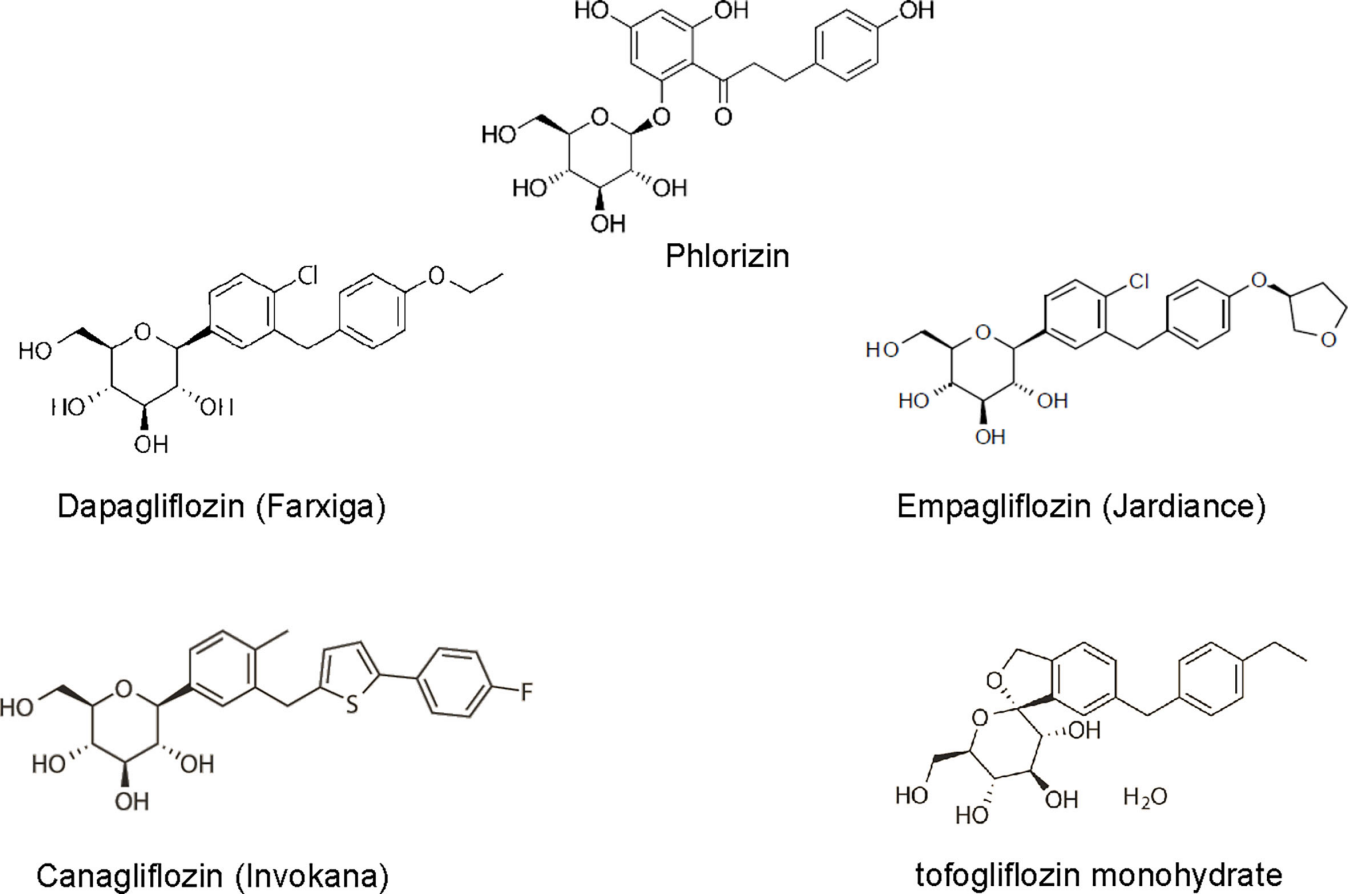
The 2-D view of chemical structure of various SGLT2 inhibitors, including phlorizin, dapagliflozin, empagliflozin, canagliflozin, tofogliflozin.

The first SGLT2 inhibitor, phlorizin, was isolated from the root bark of the apple tree in 1835 and was originally used as an antipyretic. It was 50 years later that its glycosuric effects were observed and research began to discover its mechanism of action. Phlorizin was subsequently found to be a potent but non-selective inhibitor of many isoforms of SGLT. However, due to its poor bioavailability (15%), low SGLT2 selectivity, and gastrointestinal side effects from SGLT1 blockade, phlorizin failed to progress to use in humans (7). Nevertheless, its beneficial effects for the use in diabetic patients sparked the research and development of structurally similar compounds with more selectivity towards SGLT2 and it remains the model from which all SGLT inhibitors have been developed. With advances in research, the SGLT2 inhibitors have become more selective towards the SGLT2 than SGLT1. The SGLT2 inhibitors currently approved by the FDA in the United States include dapagliflozin, canagliflozin, empagliflozin, and tofogliflozin **(** **Figure 1** **)**.

## Targeting of SGLT2 in DKD and Other Diabetic Complications

In addition to diabetes management, SGLT2 inhibitors have shown promise in the areas of chronic kidney disease (CKD), and are able to reduce uric acid levels, attenuate oxidative stress, exert anti-inflammatory actions, improve fibrosis, decrease blood pressure, decrease body weight, and mitigate hyperlipidemia, cardiovascular disease, and even some cancers, such as early-stage lung adenocarcinoma (17) and pancreatic and prostate cancer cells (18), which are saturated with SGLT2 receptors.

### Regulation of Body Fluid Homeostasis by SGLT2 Inhibitors

The glycosuric effect of SGLT2 inhibitors induces a sustained diuretic and natriuretic effect that activates compensatory mechanisms to increase fluid and food intake to stabilize body fluid volume. A study performed by Masuda et al. (19) revealed in Goto-Kakizaki rat models of T2DM that ipragliflozin induced sustained glucosuria, diuresis, and natriuresis, with compensatory increases in fluid intake and vasopressin-induced solute-free water reabsorption in proportion to the reduced fluid balance to maintain body fluid volume. Meanwhile, ipragliflozin increased renal cell membrane expression of SGLT2, aquaporin 2, and vasopressin V2 receptors. It is believed that the osmotic diuretic effect of glucose enhances water loss and causes a small increase in serum sodium concentrations which triggers vasopressin release (19). In addition, atrial natriuretic peptide (ANP) and brain natriuretic peptide (BNP) are proteins released by cardiac atria and ventricle, respectively, with hormonal properties that trigger a urinary natriuresis resulting in a reduction of volume which plays an important role in cardio-renal homeostasis. The elevated plasma level of ANP and BNP can be a marker of renal dysfunction as it occurs in CKD. SGLT2 inhibitors have been repeatedly shown by several studies to improve ANP secretion in patients with newly diagnosed T2DM, delay elevation of BNP in older patients with T2DM, and improve renal function due to reduction of BNP levels (20–22). In consistency, a preplanned subanalysis of a trial in which 120 patients with heart failure and reduced ejection fraction were randomly assigned to receive empagliflozin or placebo for 12 weeks demonstrated that empagliflozin reduced estimated extracellular volume, estimated plasma volume, and measured GFR after 12 weeks, inferring that a substantial reduction in fluid volumes might be an important mechanism underlying the cardiorenal benefits of SGLT2 inhibitors (23).

### Renal and Cardiovascular Benefits of SGLT2 Inhibitors in Diabetes

Albuminuria is one of the commonly used clinical indicators of diabetic nephropathy (DN) and is an independent and modifiable risk factor for cardiovascular events, kidney injury and mortality. In addition to blood pressure and glycemic control, the current clinical management of DKD is largely confined to the use of blockades of the renin-angiotensin-aldosterone system (RAAS), which is of limited utility with unsatisfying therapeutic efficacy (24). The advent of SGLT2 inhibitors has provided a novel and promising choice of treatment for diabetes and DKD. In the CANVAS trial, among patients with T2DM at a high risk for cardiovascular events, canagliflozin treatment was associated with a lower risk of cardiovascular events and reduced the rate of renal decline and heart failure hospitalizations compared to those who received placebo alone but, interestingly, increased the risk of amputation, primarily of the toe and/or metatarsal. The data from this study suggested that SGLT2 inhibitors are likely to exert a protective effect on cardiovascular and renal systems in diabetic patients across a wide range of albuminuria, with greater benefits in those with macroalbuminuria. There are several speculated mechanisms to explain the renoprotective action observed with SGLT2 inhibitors. First, SGLT2 inhibitors have been shown to induce an acute decrease in estimated glomerular filtration rate (eGFR) ensued by long-term preservation of kidney function, which may denote the mitigation of intraglomerular hypertension and glomerular hyperfiltration (25). Second, some have suggested that there could be changes in vascular endothelial function which may explain the improvement. And third, pre-clinical animal studies support that SGLT2 inhibitors improve kidney tissue oxygenation and exert anti-inflammatory and anti-fibrotic effects (26, 27).

In another study, a group reviewed the data from the EMPA-REG OUTCOME trial to assess the differential outcomes in kidney function by examining the slopes of eGFR changes. The study had originally shown that empagliflozin reduced the rate of cardiovascular events and slowed the progression of kidney disease among patients with T2DM and high cardiovascular risk. A review of this data showed that empagliflozin is able to significantly retard eGFR decline over about 3 years of treatment, including in patients at an increased risk for progressive renal impairment. Furthermore, the slope patterns of empagliflozin therapy revealed an action on intrarenal hemodynamics with an initial transient decline in eGFR ensued by sustained long-term preservation of renal function, reminiscent of the effects achieved with angiotensin-converting enzyme inhibitors or angiotensin II receptor blockers. After discontinuation of empagliflozin treatment, the slopes of eGFR changes demonstrated a quick upward shift toward the baseline, inferring a swift loss of the renal hemodynamic effect (28). The renal benefits associated with empagliflozin are thought to be attributable to the recovery of the tubuloglomerular feedback, resulting in a correction of intraglomerular hypertension and glomerular hyperfiltration (29).

The CREDENCE randomized trial had shown that among patients with T2DM and DN characterized by albuminuria and no more than a mild reduction in GFR, canagliflozin at the dose of 100 mg/d reduced the risk of end-stage kidney disease, doubling of serum creatinine from baseline, and death from renal or cardiovascular disease when compared to placebo. The renoprotective effects observed with this study are likely due to improved blood pressure control secondary to decreased sodium reabsorption in the PCT, thereby causing increased sodium delivery to the macula densa in the distal renal tubule and inducing afferent vasoconstriction by tubuloglomerular feedback, leading to lowered glomerular hyperfiltration (30).

In a secondary analysis of the CREDENCE trial, canagliflozin consistently and safely prevented renal and cardiovascular events in participants with substantial albuminuria across eGFR categories of 30 to <45, 45 to <60, and 60 to <90 ml/min per 1.73 m^2^. These benefits were attained on the background of universal use of renin-angiotensin system (RAS) blockades. Canagliflozin therapy also led to an acute drop in eGFR followed by a decrease in albuminuria at week 3 that was significant in every eGFR subgroup, although the drop was least in those with screening eGFR of 30 to <45 ml/min per 1.73 m^2^. Furthermore, canagliflozin led to a slower eGFR decline in every eGFR category compared with placebo, with no evidence that the benefit differed among eGFR subgroups. The findings raise important questions on whether these agents would benefit kidney disease outcomes in nondiabetic settings (31).

### Effect of SGLT2 Inhibitors on Systemic and Intrarenal RAAS

A study published in 2019 by Schork et al. investigated the effects of SGLT2 inhibitors, including empagliflozin and dapagliflozin, on body composition, fluid status, and the RAS in patients with T2DM. They found that reduction in body weight and epicardial fat was associated with a reduction in adipose tissue mass and transient loss of extracellular fluid, which was accompanied by a transient upregulation of the systemic RAS without activation of the intrarenal RAS. This bodyweight reduction can be explained by a combination of calorie deficit (due to glycosuria) and increased glycosuria resulting in increased lipid utilization and decreased triglyceride levels from gluconeogenesis induced by increased glucagon release from the pancreatic alpha-cells. Furthermore, SGLT2 inhibitors induced an osmotic diuretic effect due to increased glucosuria and natriuresis, but they did not observe an ongoing fluid loss. As a consequence of the diuretic effects, there was a reduction in arterial blood pressure after 3-6 months of treatment. SGLT2 inhibitors have also been observed to improve vascular stiffness, resulting in increased elasticity and more efficient blood pressure regulation (30). Furthermore, the initiation of SGLT2 inhibitors was shown to induce a short-term elevated natriuresis followed by a compensatory increase in sodium reuptake through tubular transporters and transient systemic activation of the RAAS in patients with T2DM (32). Interestingly, the intrarenal RAAS was not activated. In this study, despite pre-existing treatment with RAAS inhibitors, nearly all patients experienced an increase in renin and aldosterone activity after 30 days with normalization after 6 months of treatment. It is still unclear how and why SGLT2 inhibitors activate the systemic RAS but not the intrarenal RAS during the early stages of treatment in T2DM. Volume loss and/or sodium depletion likely explains the systemic RAAS activation, although there may be other contributors involved that are not yet accounted for. As far as the intrarenal RAAS not being activated, it is speculated that angiotensinogen may be involved as it is expressed in the proximal tubules, and treatment with SGLT2 inhibitors appear to affect intrarenal angiotensinogen production possibly due to changes in glucose levels which have been shown to decrease angiotensinogen production in the early PCT (33). However, this is all actively being researched as there are no clear answers at present.

### Inhibitory Effect of SGLT2 Inhibitors on Renal Oxidative Stress in DKD

Oxidative stress is highly associated with the pathophysiology of CKD and DN. Oxidative stress develops when the production of free radicals overcomes the capacity of the anti-oxidant defense system, resulting in damage to various biological elements. It is posited that oxidative stress induces renal injury through modulation of transcription factors, induction of inflammatory responses, enhancement of advanced glycation end product (AGE) and the receptor for advanced glycation end product (RAGE) production, upregulation of protein kinase C (PKC), and by direct modification of intracellular molecules (34). Dapagliflozin has been shown to slow DN progression by diminishing the production of free radical progenitors (35). Ipragliflozin has been shown to normalize glucose metabolism and re-adjust the oxidative balance in the kidneys of diabetic animals (36). In diabetic rats, phlorizin prevented oxidative stress by promotion of catalase and glutathione peroxidase activity and reduced nitrogen free radicals (37). Moreover, empagliflozin has been shown to partly suppress oxidative stress in DN *via* suppression of the AGE-RAGE axis (38).

### Anti-Inflammatory Effect of SGLT2 Inhibitors in DKD

CKD is accompanied by the progression of higher circulating levels of inflammatory mediators and fibrogenesis. DN has been associated with the upregulation of myriad inflammatory cytokines including IL-1, IL-6, IL-18, TNF-α, ICAM, VCAM, and MCP-1 (39). SGLT2 inhibitors are able to reduce serum leptin and IL-6 levels and increase adiponectin concentrations, resulting in improved adipose tissue function and decreased tissue inflammation (30). *In vitro*, in cultured human proximal tubular cells exposed to high ambient glucose, empagliflozin was able to mitigate the expression of inflammatory/fibrotic markers including IL-6 and collagen IV, consistent with an anti-inflammatory and anti-fibrotic activity (40). Diabetic animals treated with dapagliflozin were also shown to have a dose-dependent reduction in renal inflammation and fibrosis (41). Uric acid is closely associated with inflammatory biomarkers and is *per se* a damage-associated molecular pattern (DAMP), that is able to activate the NLRP3 inflammasome and cause inflammation. An elevated plasma uric acid level can independently predict the development of CKD and has been associated with the progression towards renal failure (42). SGLT2 inhibitors cause increase urinary excretion of uric acid by altering its tubular transport along the apical membrane of the collecting ducts. Canagliflozin, dapagliflozin, and empagliflozin have all been associated with increased urinary excretion of uric acid (43–47).

### Effect of SGLT2 Inhibitors on Metabolic Reprogramming in Diabetes

**Figure 2:**
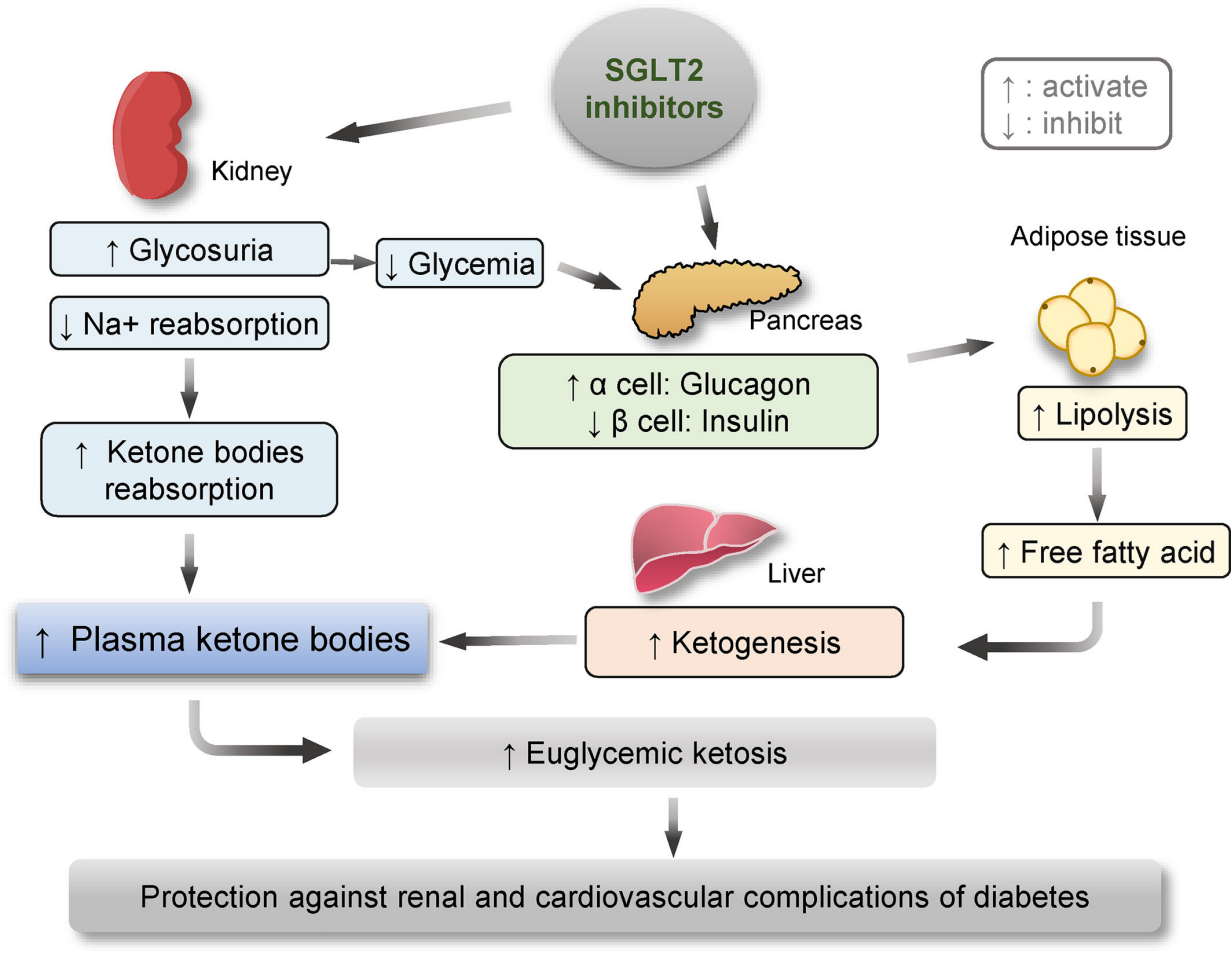
Schematic diagram depicting the metabolic reprogramming subsequent to therapeutic targeting of SGLT2, including enhanced ketogenesis, lipolysis and glucagon effects. One of the major actions of SGLT2 inhibitors is to block SGLT2 in renal proximal tubular epithelial cells and thus reduce renal reabsorption of sodium and glucose, leading to glycosuria and normalized glycemic levels in patients with diabetes. The glycosuric effect of SGLT2 inhibitors is associated with increased renal reabsorption of ketone bodies, contributing to an elevated plasma level of ketone bodies. On the other hand, SGLT2 inhibitors-reduced glycemic levels may augment the pancreatic release of glucagon and decrease insulin production, which together act on fat tissue and promote lipolysis. Amplified levels of free fatty acid in blood along with reduced glycemic levels may reinforce ketogenesis in the liver, ultimately resulting in ketosis, which has been lately demonstrated to confer a protective effect on the kidney and the cardiovascular system in both diabetic and non-diabetic kidney disease.

In addition, recent evidence indicates that SGLT2 inhibitors may induce metabolic reprogramming, which may be responsible for their cardiorenal benefits in diabetes (**Figure 2**). In a recent study performed by Bonner et al., the expression of SGLT2 transporters was probed on pancreatic α cells that are responsible for glucagon secretion. They confirmed that, in human and rodent pancreatic α-cells, glucagon secretion markedly increased when glucose concentrations were below physiological levels. In healthy mice, bacterial infections treatment promoted glucagon secretion and hepatic gluconeogenesis limiting the decrease of plasma glucose due to glycosuria during the fasting state. In patients with T2DM, dapagliflozin treatment increased both plasma glucagon and endogenous glucose production, though dapagliflozin-treated patients had lower blood glucose levels than those receiving placebo due to glycosuria. Their results suggest that sodium-glucose co-transport by SGTL2 is essential for the appropriate regulation of glucagon secretion when glucose concentrations are within the physiological range. This direct effect of SGLT2 inhibition on α cells likely offsets the glucose-lowering effects of dapagliflozin and help explain why this medication is unlikely to induce hypoglycemia (9).

Moreover, SGLT2 inhibitors-induced glycosuria is associated with a constellation of signs of metabolic adaptation, including reinforced ketogenesis and elevated β-hydroxybutyrate (β-HB) levels (48). The metabolic state of ketosis is a physiological response to starvation, during which, the carbohydrate stores in the body are rapidly depleted, and the body uses fat reserves instead. Some fat is converted by the liver to ketone bodies, providing an alternative fuel source (49, 50). More and more studies suggest that ketone bodies may convey a salutary action on aging and a number of chronic diseases, like obesity, diabetes, and CKD. Mechanistically, in addition to serving as an alternative fuel substrate, ketone bodies like β-HB, which is the predominant component of ketone bodies, are potent regulators of cellular signaling pathways including mTOR, AMPK, and HDACs and intercept multiple pathological processes, such as metabolic reprogramming, NLRP3 inflammasome, and pyroptosis, some of which have been implicated in kidney diseases. Indeed, the lastest evidence demonstrates that β-HB exerts a protective effect in glomerular podocytes against diabetes-elicited senescence response (51), which plays a key role in diabetes-accelerated kidney aging and degeneration (52). Thus, the ketogenic effect of SGLT2 inhibitors seems to provide extra benefits for patients with cardiovascular and renal complications of diabetes. Indeed, while the glucose-lowering capacity of empagliflozin is similar to sulfonylurea in patients with T2DM, empagliflozin shows a greater reduction in IL-1β secretion compared to sulfonylurea accompanied by decreased serum insulin and increased serum β-HB (53). A recent meta-analysis demonstrated that SGLT2 inhibitor therapy is associated with a remarkable decrease in the risk of cardiovascular events and renal impairment (54). Provided the protective effect of β-HB against diabetic kidney injury, it is tempting to conceive that the renoprotective activity of SGLT2 inhibitors may be mediated, at least in part, by β-HB.

## Potential Concerns With Clinical Use of SGLT2 Inhibitors in Diabetes

Up to this moment, SGLT2 inhibitors have been shown to be safe and beneficial for use in patients with a GFR ≥30mL/min/1.73m^2^ with or without RAS blockade. There is strong evidence from the clinical trials that SGLT2 inhibitors should be prioritized in patients with T2DM and CKD with a presenting GFR of 30-40mL/min/1.73m^2^. The CEDENCE trial suggested that the combined use of SGLT2 inhibitors and RAS blockade should be well tolerated from a hemodynamic perspective as no participants were at increased risk of developing AKI, volume depletion, or hyperkalemia. Most importantly, in the CREDENCE trial, participants whose eGFR fell to <30mL/min/1.73m^2^ were continued on randomized treatment until dialysis or transplantation. As a result, the FDA now permits the continued use of canagliflozin with an eGFR <30mL/min/1.73m^2^ until dialysis or transplantation in patients already initiated on therapy (55). Another concern for the use of SGLT2 inhibitors is its use along with a loop diuretic, as diuretic use becomes increasingly common as kidney function declines. In patients with unstable volume status, it is recommended that SGLT2 inhibitors should not be initiated along with loop diuretics or should be used along with a decreased dose of loop diuretic and close monitoring (56).

Currently, the use of SGLT2 inhibitors is limited due to the high cost. Along with their increasing use, there is also the concern for serious, if not fatal, infections that have deterred most physicians from initiating therapy. Indeed, SGLT2 inhibitors-induced glycosuria makes the genital area more conducive to bacterial infections, and life-threatening infection of the genitals and areas around the genitals, such as necrotizing fasciitis of the perineum* *or* *Fournier’s gangrene, has been reported (57). In addition, urinary tract and vaginal yeast infections have also been associated with the use of SGLT2 inhibitors (58). However, serious infection associated with SGLT2 inhibitor use is rare. Thus, this concern should not deter its use in patients without lower extremity wounds or recurrent urinary tract infection as long as proper monitoring is in place. We will learn more about the benefits of SGLT2 inhibitors as upcoming and ongoing trials will provide conclusive and definitive data on cardiovascular and renal protection in DKD patients. These include the DAPA-CKD study for dapagliflozin (59), the EMPA-KIDNEY study for empagliflozin (60), the SCORED study for sotagliflozin (61), the VERTIS study (62), and the ongoing DIAMON study (63). Some of these trials will also enroll patients with eGFR as low as 20 ml/min per 1.73 m^2^, regardless of the magnitude of albuminuria (24, 31). Of further note, although this was not observed in any of the studies discussed here, SGLT2 inhibitors have been suspected to increase the occurrence of stroke and pre-renal acute kidney injury due to volume depletion, but as long as counter-regulatory mechanisms are functioning, this does not appear to pose any real threat (32).

## Conclusions and Perspectives

Diabetes-associated kidney disease is a major precipitating factor resulting in renal replacement therapy. The advent of SGLT2 inhibitors has provided a major advance for the prevention and treatment of T2DM, CKD, and cardiovascular events. Beyond the glycosuric effects, SGLT2 inhibitors have exhibited renal protective properties, which are associated with improved glycemic control, improved blood pressure/hemodynamics, weight loss, prevention of oxidative stress, anti-inflammatory properties, anti-fibrotic processes, reduction in albuminuria, reduction in intrarenal RAS activation, antioxidant and anti-inflammatory effects, reduction in plasma uric acid levels, metabolic reprogramming and reduction in natriuretic peptide levels. Despite concerns over the safety issues related to serious but rare infective complications, clinical use of SGLT2 inhibitors in patients with DKD seems to be effective, safe, and well-tolerated, resulting in a unique renoprotective action and cardiovascular benefits. Future research efforts are merited to understand the exact molecular mechanism responsible for the beneficial activity of SGLT2 inhibitors in diabetic patients, to reveal the most suitable diabetic patients to receive SGLT2 inhibitor treatment, and to test the potential of SGLT2 inhibitors in improving non-diabetic CKD in men.

## Author Contributions

RG devised the conceptual ideas. JS performed the literature search and drafted the original manuscript. BC drew the figures. LD, DM, and RG contributed to revision. All authors agreed that the entire concept and ownership of this work belong to RG. All authors contributed to the article and approved the submitted version.

## Funding

The research work was supported in part by the Research Incentive Fund from the University of Toledo. R. Gong was supported by the U.S. National Institutes of Health grant DK114006. The funders had no role in the design and conduct of this study, collection and interpretation of the data, or preparation and approval of the manuscript.

## Conflict of Interest

The authors declare that the research was conducted in the absence of any commercial or financial relationships that could be construed as a potential conflict of interest.

## Publisher’s Note

All claims expressed in this article are solely those of the authors and do not necessarily represent those of their affiliated organizations, or those of the publisher, the editors and the reviewers. Any product that may be evaluated in this article, or claim that may be made by its manufacturer, is not guaranteed or endorsed by the publisher.
